# Chronic Intra-Uterine *Ureaplasma parvum* Infection Induces Injury of the Enteric Nervous System in Ovine Fetuses

**DOI:** 10.3389/fimmu.2020.00189

**Published:** 2020-03-17

**Authors:** Cathelijne Heymans, Ilse H. de Lange, Matthias C. Hütten, Kaatje Lenaerts, Nadine J. E. de Ruijter, Lilian C. G. A. Kessels, Glenn Rademakers, Veerle Melotte, Werend Boesmans, Masatoshi Saito, Haruo Usuda, Sarah J. Stock, Owen B. Spiller, Michael L. Beeton, Matthew S. Payne, Boris W. Kramer, John P. Newnham, Alan H. Jobe, Matthew W. Kemp, Wim G. van Gemert, Tim G. A. M. Wolfs

**Affiliations:** ^1^Department of Surgery, NUTRIM School of Nutrition and Translational Research in Metabolism, Maastricht University, Maastricht, Netherlands; ^2^Department of Pediatrics, School for Oncology and Developmental Biology (GROW), Maastricht University, Maastricht, Netherlands; ^3^Neonatology, Department of Pediatrics, Maastricht University Medical Center, Maastricht, Netherlands; ^4^Neonatology, Department of Pediatrics, University Hospital Aachen, Aachen, Germany; ^5^Neonatology, Department of Pediatrics, University Children's Hospital Würzburg, Würzburg, Germany; ^6^Department of Pathology, School for Oncology and Developmental Biology (GROW), Maastricht University Medical Center, Maastricht, Netherlands; ^7^Biomedical Research Institute (BIOMED), Hasselt University, Hasselt, Belgium; ^8^Division of Obstetrics and Gynecology, University of Western Australia, Perth, WA, Australia; ^9^Center for Perinatal and Neonatal Medicine, Tohoku University Hospital, Sendai, Japan; ^10^Usher Institute, University of Edinburgh, Edinburgh, United Kingdom; ^11^Division of Infection and Immunity, School of Medicine, Cardiff University, Cardiff, United Kingdom; ^12^Cardiff School of Sport and Health Sciences, Cardiff Metropolitan University, Cardiff, United Kingdom; ^13^Division of Neonatology/Pulmonary Biology, The Perinatal Institute, Cincinnati Children's Hospital Medical Center, University of Cincinnati, Cincinnati, OH, United States; ^14^School of Veterinary and Life Sciences, Murdoch University, Perth, WA, Australia; ^15^Pediatric Surgery, Department of Surgery, Maastricht University Medical Center, Maastricht, Netherlands; ^16^Department of Surgery, University Hospital Aachen, Aachen, Germany; ^17^Department of Biomedical Engineering (BMT), School for Cardiovascular Diseases (CARIM), Maastricht University, Maastricht, Netherlands

**Keywords:** *Ureaplasma parvum*, intra-amniotic infection, chorioamnionitis, enteric nervous system, sheep, preterm birth, necrotizing enterocolitis

## Abstract

**Background:** Chorioamnionitis, inflammation of the fetal membranes during pregnancy, is often caused by intra-amniotic (IA) infection with single or multiple microbes. Chorioamnionitis can be either acute or chronic and is associated with adverse postnatal outcomes of the intestine, including necrotizing enterocolitis (NEC). Neonates with NEC have structural and functional damage to the intestinal mucosa and the enteric nervous system (ENS), with loss of enteric neurons and glial cells. Yet, the impact of acute, chronic, or repetitive antenatal inflammatory stimuli on the development of the intestinal mucosa and ENS has not been studied. The aim of this study was therefore to investigate the effect of acute, chronic, and repetitive microbial exposure on the intestinal mucosa, submucosa and ENS in premature lambs.

**Materials and Methods:** A sheep model of pregnancy was used in which the ileal mucosa, submucosa, and ENS were assessed following IA exposure to lipopolysaccharide (LPS) for 2 or 7 days (acute), *Ureaplasma parvum* (UP) for 42 days (chronic), or repetitive microbial exposure (42 days UP with 2 or 7 days LPS).

**Results:** IA LPS exposure for 7 days or IA UP exposure for 42 days caused intestinal injury and inflammation in the mucosal and submucosal layers of the gut. Repetitive microbial exposure did not further aggravate injury of the terminal ileum. Chronic IA UP exposure caused significant structural ENS alterations characterized by loss of PGP9.5 and S100β immunoreactivity, whereas these changes were not found after re-exposure of chronic UP-exposed fetuses to LPS for 2 or 7 days.

**Conclusion:** The *in utero* loss of PGP9.5 and S100β immunoreactivity following chronic UP exposure corresponds with intestinal changes in neonates with NEC and may therefore form a novel mechanistic explanation for the association of chorioamnionitis and NEC.

## Introduction

Preterm birth is a common and major worldwide health issue, contributing to significant neonatal morbidity and mortality (1). Around 1 in every 10 births are preterm, accounting for ~15 million premature newborns each year (2). Due to complications, over one million premature newborns will die shortly after birth (3, 4). Chorioamnionitis, defined as inflammatory cell infiltration of fetal membranes, is frequently associated with preterm birth and typically occurs due to an ascending bacterial infection (5–7) that can be acute or chronic (8). Intrauterine exposure of preterm infants to chorioamnionitis is associated with an increased risk of adverse neonatal outcomes (9, 10), including necrotizing enterocolitis (NEC) (9, 11, 12). Adverse gastrointestinal outcomes have been associated with both systemic fetal inflammatory response syndrome (FIRS) and direct exposure of the gut to the swallowed infected amniotic fluid (11, 13, 14). Chorioamnionitis can occur with intact membranes, which is common for genital mycoplasmas, such as *Ureaplasma* species (spp.), present in the lower genital tract of women (6, 15). *Ureaplasma* spp. can cause chronic chorioamnionitis that does not evoke a maternal response, but is still associated with adverse fetal outcomes (16). In an experimental large animal model, we previously showed that a *Ureaplasma parvum* (UP) serovar 3 infection up to 14 days prior to delivery causes fetal gut inflammation with damaged villus epithelium, gut barrier loss, and severe villus atrophy (17).

The injury caused by intrauterine *Ureaplasma* spp. exposure might derive from the direct inflammatory reaction, as well as from potential interactions with other inflammatory stimuli. Chorioamnionitis is often polymicrobial, as over 65% of positive amniotic fluid cultures lead to the identification of two or more pathogens (7). In this context, we previously showed that cerebral and lung immune activation following intra-amniotic (IA) lipopolysaccharide (LPS) exposure was prevented when these animals were chronically pre-exposed to UP serovar 3 (18, 19). This illustrates that interactions between different microbes can occur, leading to organ-specific sensitization or preconditioning.

The enteric nervous system (ENS) consists of enteric neurons and glial cells, autonomously regulates gastrointestinal activity (i.e., secretion, absorption, and motility), and contributes to gut integrity (20). The formation of the ENS requires coordinated migration, proliferation, and differentiation of neural crest progenitors, directed neurite growth, and establishment of a network of interconnected neurons and glia (21, 22). Although these processes mostly occur *in utero*, an important part of ENS development takes place postnatally (23, 24). Neonates with NEC have structural and functional damage of the submucosal and myenteric plexus, including loss of enteric neurons and glial cells (25–27). The involvement of chorioamnionitis in the induction of adverse intestinal outcomes including NEC, combined with the presence of ENS abnormalities in NEC, prompted us to study the impact of an antenatal infection on the ENS.

The aim of this study was therefore to investigate the effect of acute IA exposure to LPS and chronic exposure to UP on the intestinal mucosa and ENS in fetal lambs using a well-established sheep model of chorioamnionitis. In addition, we investigated the potential interactions of repetitive IA microbial stimuli by acute exposure to LPS in ovine fetuses that were chronically pre-exposed to UP.

## Materials and Methods

### Animal Model and Experimental Procedures

All experiments were approved by the animal ethics committee of the University of Western Australia (Perth, Australia).

**Figure 1:**
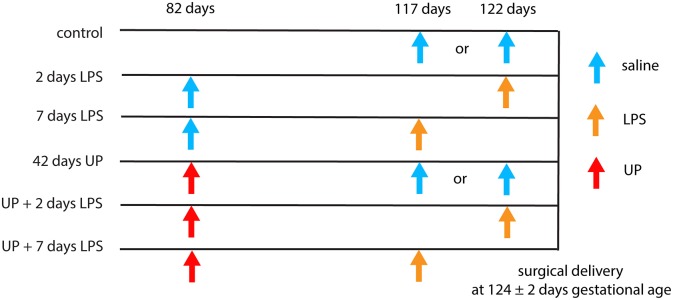
Different intervention study groups. All injections were delivered by ultrasound-guided amniocentesis. Timing is shown in gestational days.

The animal model and experimental procedures were previously described (18). Briefly, 39 date-mated merino ewes were randomly assigned to six different groups of between five and eight animals to receive IA injections under ultrasound guidance. Verification of the IA injections was done by amniotic fluid electrolyte analysis. The date-mated pregnant ewes received an IA injection of an *in vitro* cultured strain HPA5 of UP serovar 3 (2 × 10^5^ color-changing units, CCU) (28) 42 days prior to delivery (at 82 days of gestation, which corresponds to the second trimester in humans) or *Escherichia coli*-derived LPS (O55:B5; Merck, Darmstadt, Germany), 10 mg in 2 ml of saline, 2 or 7 days prior to delivery (at respectively, 122 and 117 days of gestation). Previously, we have shown that the half-life time of LPS in the amniotic fluid is relatively long (1.7 days) and that the LPS amount is higher than the essential threshold of 1 mg for at least 5 days (29, 30). Chronic sustained UP infection was confirmed by positive culture of amniocentesis samples at intermediate time points and sterile amniotic fluid samples collected at cesarean delivery, as previously described (31). Two or seven days LPS exposure (prior to cesarean delivery) represents an acute inflammatory challenge. To evaluate the combined effect between these inflammatory modalities, a subgroup of chronically UP-infected ewes received IA LPS at 35 and 40 days post-UP infection (i.e., 7 or 2 days LPS exposure prior to delivery following 42 days of UP infection). A group receiving IA injections of sterile saline (2 or 7 days prior to delivery, respectively six and two animals which were pooled) served as controls (Figure 1). Fetuses were surgically delivered at 124 ± 2 days of gestational age (term gestation in sheep = 150 days), equivalent of ~30 weeks of human gestation. After delivery, fetuses were euthanized with intravenous pentobarbitone (100 mg/kg). For this experiment, fetuses of both sexes were used.

### Sampling

During necropsy, blood and terminal ileum samples were collected. Ileum samples were fixed in 10% formalin and embedded in paraffin, or snap frozen in liquid nitrogen. Where insufficient paraffin-embedded material was available for study, additional material was generated by paraffin embedding snap-frozen tissue samples: frozen tissue blocks were defrosted, fixed in 4% formaldehyde at room temperature overnight, and transferred to 70% ethanol prior to embedding in paraffin with the use of a vacuum infiltration processor.

### Antibodies

The following antibodies were used for immunohistochemistry: polyclonal rabbit anti-myeloperoxidase (MPO; A0398, Dakocytomation, Glostrup, Denmark) for the identification of neutrophils, polyclonal rabbit anti-cluster of differentiation 3 (CD3; A0452, Dakocytomation) for the detection of T cells, polyclonal rabbit anti-bovine protein gene product 9.5 (PGP9.5; Z5116, Dakocytomation) for the detection of enteric neurons, polyclonal rabbit anti-doublecortin (Ab18723, Abcam, Cambridge, UK) for the detection of immature neurons, polyclonal rabbit anti-glial fibrillary acidic protein (GFAP; Zo334, Dakocytomation) to detect enteric glial cell reactivity/activation (32), and polyclonal rabbit anti-S100β (PA5-16257, Invitrogen, Carlsbad, CA, USA) as a marker for enteric glial cells.

The following secondary antibodies were used: peroxidase-conjugated polyclonal goat anti-rabbit (111-035-045, Jackson, West Grove, PA, USA) (MPO), peroxidase-conjugated polyclonal swine anti-rabbit (P0399, DakoCytomation) (doublecortin) and BrightVision+ Poly-HRP-Anti Mouse/Rabbit IgG biotin-free (ImmunoLogic, Duiven, Netherlands) (PGP9.5), and biotin-conjugated polyclonal swine anti-rabbit (E0353, DakoCytomation) (CD3, GFAP, S100β).

### Immunohistochemistry

Formalin-fixed terminal ileum was embedded in paraffin and 4 μm sections were cut. After deparaffinization and rehydration, endogenous peroxidase activity was blocked with 0.3% H_2_O_2_ diluted in phosphorylated buffer saline (PBS, pH 7.4). Antigen retrieval was performed with citrate buffer for CD3, PGP9.5, doublecortin, and S100β stainings. Non-specific binding was blocked for 30 min at room temperature with 10% normal goat serum (NGS) in PBS (MPO), 5% NGS in PBS (doublecortin), or 5% bovine serum albumin (BSA) in PBS (CD3, GFAP, and S100β). For PGP9.5, non-specific binding was blocked for 10 min at room temperature with 20% fetal calf serum (FCS). Thereafter, sections were incubated with the primary antibody of interest and subsequently incubated with the respective secondary antibody. MPO, PGP9.5, and doublecortin were detected by using a peroxidase-conjugated secondary antibody and antibodies against CD3, GFAP, and S100β were detected with avidin–biotin complex (Vectastain Elite ABC kit, Bio-connect, Huissen, Netherlands). Substrate staining was performed for MPO with 3-amino-9-ethylcarbazole (AEC; Merck, Darmstadt, Germany). Immunoreactivity for CD3 and GFAP was detected by using nickel-DAB. Immunoreactivity for PGP9.5, doublecortin, and S100β was detected by using DAB. Hematoxylin (MPO, PGP9.5, doublecortin, and S100β) or nuclear fast red (CD3 and GFAP) was used as a counterstain for nuclei.

### Qualitative Analysis of Damage of the Terminal Ileum

H&E slides were analyzed by two independent investigators blinded to the experimental setup to assess damage of the terminal ileum. A scoring system from 0 to 4 was used to describe the severity of histological injury. Scoring was as follows: 0, no damage; 1, disrupted epithelial lining, but no loss of enterocytes; 2, disrupted epithelial lining, mild enterocyte loss from the villus tips; 3, disrupted epithelial lining, moderate enterocyte loss from villus tips, some debris in the lumen; and 4, abundant enterocyte loss from villus tips, abundant debris in the lumen, and severe shedding of villus tips.

### Quantification of Immunohistochemical Stainings

The stained tissue sections were scanned with the Ventana iScan HT slide scanner (Ventana Medical Systems, Oro Valley, AZ, USA). Of these images, viewed with Panoramic Viewer (version 1.15.4, 3DHISTECH, Budapest, Hungary), random images of regions of interest were taken (×200).

Two investigators blinded to the study groups counted the MPO- and CD3-positive cells in three to five non-overlapping high-power fields in the mucosa and submucosa. The average MPO- and CD3-positive cells per area are reported for each animal. The percentage of area in the submucosal and myenteric ganglia positively stained for PGP9.5, doublecortin, GFAP, and S100β was determined in five non-overlapping high-power fields using Leica QWin Pro software (version 3.4.0, Leica Microsystems, Mannheim, Germany) by an investigator blinded to the study groups. Relative area staining was calculated by dividing the positively stained areas of the ganglia of the submucosal or myenteric plexus by the total area of the muscle layer. The data are expressed as fold increase over the control value.

### RNA Extraction and Quantitative Real-Time PCR

RNA was extracted from snap-frozen terminal ileum tissue using TRI reagent (Invitrogen)/chloroform extraction. Isolated RNA was DNase treated to remove possible contamination with genomic DNA by using the RQ1 RNase-Free DNase kit (Promega, Madison, WI, USA) and afterwards reverse transcribed into cDNA using oligo(dT)12–18 primers (Invitrogen) and Moloney murine leukemia virus (M-MLV) reverse transcriptase (Invitrogen). Quantitative real-time PCR (qPCR) reactions were performed with a LightCycler 480 Instrument (Roche Applied Science, Basel, Switzerland) using the SensiMix™ SYBR® No-ROX kit (Bioline, London, UK) for 45 cycles. The mRNA levels of *IL-1*β, *IL-6, IL-10*, tumor necrosis factor alpha (*TNF-*α), and interleukin-1 receptor-associated kinase 3 (*IRAK3*) were determined to assess inflammation of the terminal ileum. The mRNA levels of neuronal nitric oxide synthase (*nNOS*) and choline acetyltransferase (*CHAT*) were determined to assess the motility signaling functions of the ENS using LinRegPCR software (version 2016.0, Heart Failure Research Center, Academic Medical Center, Amsterdam, Netherlands). The geometric mean of the mRNA levels of three reference genes [ribosomal protein S15 (*RPS15*), glyceraldehyde 3-phosphate dehydrogenase (*GAPDH*), and peptidylprolyl isomerase A (*PPIA*)] were calculated and used as a normalization factor. The data are expressed as fold increase over the control value. The primer sequences are shown in Table 1.

**Table 1:** Primer sequences.

**Primer**	**Forward**	**Reverse**
RPS15	5′-CGAGATGGTGGGCAGCAT-3′	5′-GCTTGATTTCCACCTGGTTGA-3′
GAPDH	5′-GGAAGCTCACTGGCATGGC-3′	5′-CCTGCTTCACCACCTTCTTG-3′
PPIA	5′-TTATAAAGGTTCCTGCTTTCACAGAA-3′	5′-ATGGACTTGCCACCAGTACCA-3′
IL-1β	5′-AGAATGAGCTGTTATTTGAGGTTGATG-3′	5′-GTGAGAAATCTGCAGCTGGATGT-3′
IL-6	5′-ACATCGTCGACAAAATCTCTGCAA-3′	5′-GCCAGTGTCTCCTTGCTGTTT-3′
IL-10	5′-CATGGGCCTGACATCAAGGA-3′	5′-CGGAGGGTCTTCAGCTTCTC-3′
TNF-α	5′-GCCGGAATACCTGGACTATGC-3′	5′-CAGGGCGATGATCCCAAAGTAG-3′
IRAK3	5′-AGTGTGTAGGTAACACAGCCC-3′	5′-TGCTGGTCATGCTTATGGCA-3′
nNOS	5′-CGGCTTTGGGGGTTATCAGT-3′	5′-TTGCCCCATTTCCACTCCTC-3′
CHAT	5′-CCGCTGGTATGACAAGTCCC-3′	5′-GCTGGTCTTCACCATGTGCT-3′

### Data Analysis

Data are presented as median with interquartile range. Statistical analyses were performed using GraphPad Prism (version 6.01, GraphPad Software Inc., La Jolla, CA, USA). A non-parametric Kruskal–Wallis test followed by Dunn's *post hoc* test was used to analyze statistically significant group differences. Differences were considered statistically significant at *p* < 0.05. Given the relatively small animal numbers per group, we also reported actual *p* values between *p* ≥ 0.05 and *p* < 0.10 and interpreted these as potentially biologically relevant. This assumption will decrease the chance of a type II error, but increases the chance of a type I error.

## Results

### Intestinal Damage and Inflammation in the Terminal Ileum Due to Chorioamnionitis

**Figure 2:**
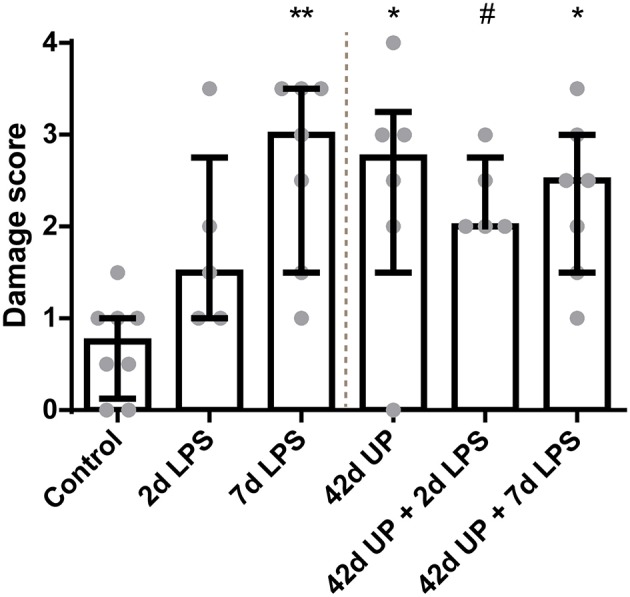
Increased mucosal injury in all groups, except for the animals exposed to 2 days LPS. ^#^*p* = 0.06, *p < 0.05, **p < 0.005 compared to the control.

There was a higher intestinal damage score for all experimental groups compared to the control (*p* < 0.005 for the 7 days LPS group, *p* < 0.05 for the 42 days UP group and 42 days UP + 7 days LPS group, and *p* = 0.06 for the 42 days UP + 2 days LPS group all compared to the control; Figure 2), except for the animals exposed to 2 days of LPS. Pre-exposure with UP did not augment mucosal injury in the LPS-treated groups.

**Figure 3:**
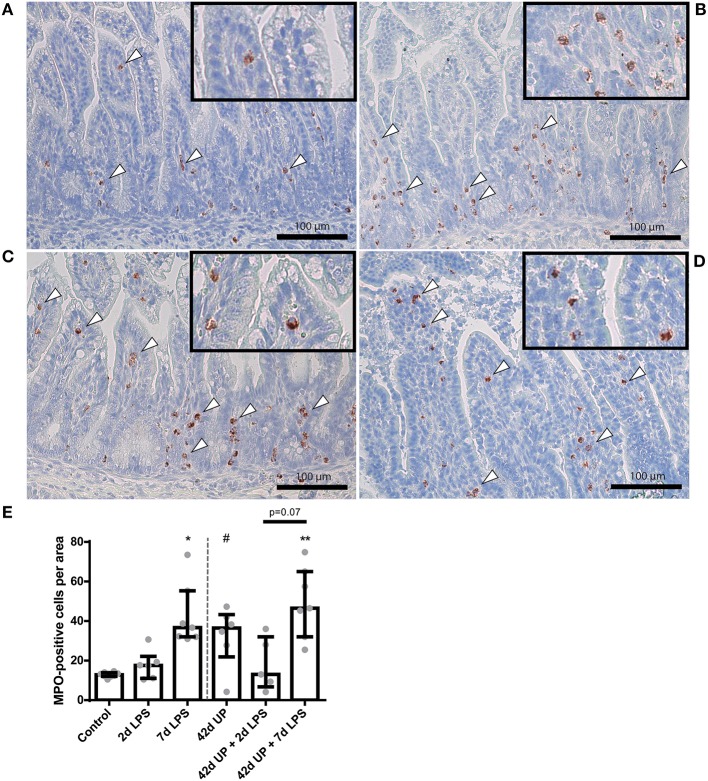
Representative images of mucosal neutrophil influx reflected by myeloperoxidase (MPO)-positive cell (indicated by white triangles) counts of the control **(A)**, 7 days lipopolysaccharide (LPS) **(B)**, *Ureaplasma parvum* (UP) **(C)**, and UP prior to 7 days LPS groups **(D)**. **(E)** Increased MPO count in animals exposed to 7 days LPS, UP, and UP prior to 7 days LPS. ^#^*p* = 0.08, *p < 0.01, **p < 0.005 compared to the control.

A statistically significant increase in MPO-positive cells was seen in the mucosa 7 days after LPS exposure compared to the control (*p* < 0.05; Figure 3). Chronic UP infection also caused an elevation of mucosal MPO-positive cells compared to the control (*p* = 0.08; Figure 3). Furthermore, combining these two inflammatory insults resulted in an increased mucosal MPO-positive cell count compared to the control (*p* < 0.005; Figure 3), and this experimental group tended to be increased when compared to the UP + 2 days LPS-exposed group (*p* = 0.07; Figure 3). LPS exposure 2 days prior to delivery was insufficient to induce mucosal MPO-positive cell infiltration. Pre-exposure to UP in combination with LPS administration did not alter the number of mucosal MPO-positive cells compared to LPS alone, both after 2 and 7 days.

**Figure 4:**
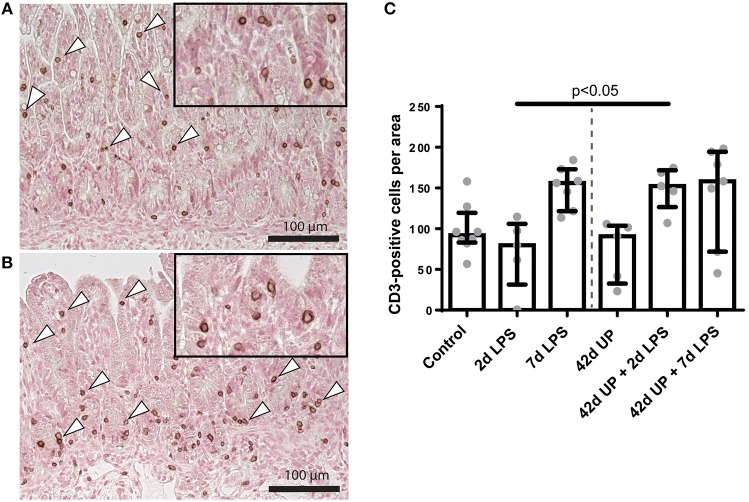
Representative images of mucosal T cell influx reflected by CD3-positive cell (indicated by white triangles) counts of the control **(A)** and *Ureaplasma parvum* (UP) prior to 2 days lipopolysaccharide (LPS) **(B)**. **(C)** Increased CD3 count in animals exposed to UP prior to 2 days LPS compared to the 2 days LPS group.

While chronic UP infection and acute LPS exposure 2 days pre-delivery did not have any effect on mucosal CD3-positive T cell presence, those animals receiving LPS 7 days pre-delivery (both uninfected and chronic UP-infected groups) as well as chronic UP-infected animals receiving LPS 2 days pre-delivery all showed apparent elevated levels of CD3-positive T cell infiltration (Figure 4). However, the only comparison to achieve *p* < 0.05 significance was that of uninfected and chronic UP-infected animals receiving LPS 2 days pre-delivery (Figure 4).

**Figure 5:**
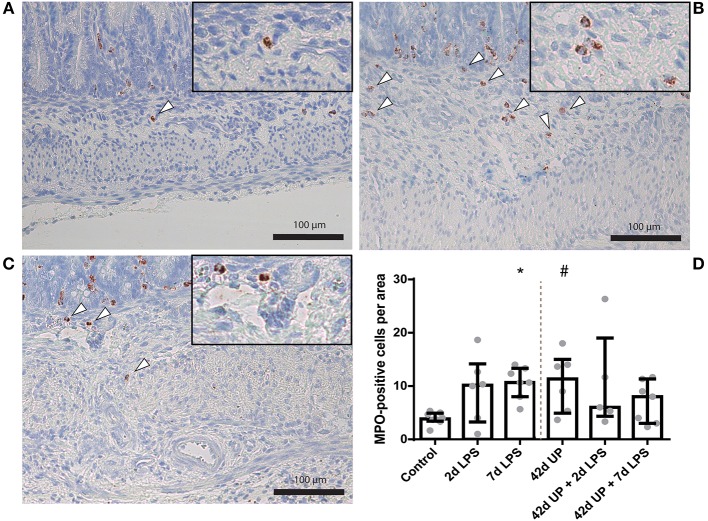
Representative images of submucosal neutrophil influx reflected by myeloperoxidase (MPO)-positive cell (indicated by white triangles) counts of the control **(A)**, 7 days lipopolysaccharide (LPS) **(B)** and *Ureaplasma parvum* (UP) **(C)**. **(D)** Increased MPO count in animals exposed to 7 days LPS and UP. ^#^*p* = 0.06, *p < 0.05 compared to the control.

For investigation of submucosal inflammation, there was an increase of MPO-positive cells in the 7 days LPS group and submucosal MPO-positive cells tended to be increased in the chronic UP infection group compared to the control (*p* < 0.05 and *p* = 0.06; Figure 5). Additional acute LPS insult (2 or 7 days pre-delivery) in chronic UP-infected animals resulted in increased variability and loss of significance in the MPO cell infiltration compared to 2 or 7 days of LPS alone.

**Figure 6:**
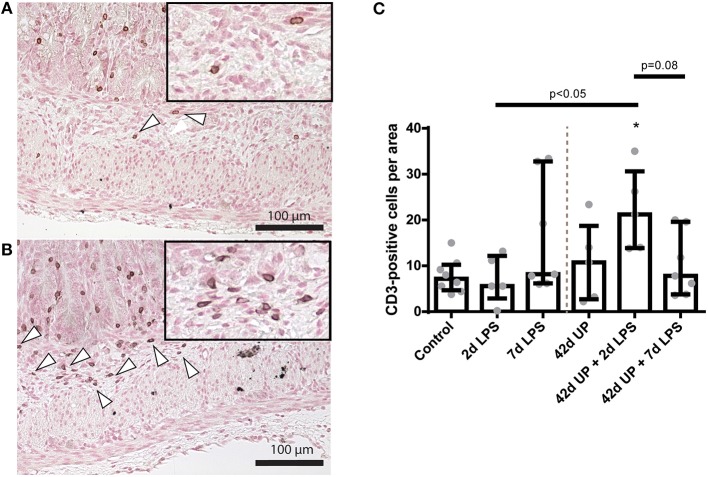
Representative images of submucosal T cell influx reflected by CD3-positive cell (indicated by white triangles) counts of the control **(A)** and *Ureaplasma parvum* (UP) prior to 2 days lipopolysaccharide (LPS) groups **(B)**. **(C)** Increased CD3 count in animals exposed to UP prior to 2 days LPS. *p < 0.05 compared to the control.

The greatest increase of submucosal CD3-positive cells was observed in 2 day LPS-exposed chronic UP-infected animals, which was significantly increased compared to the control or acute 2 day LPS stimulation alone (both *p* < 0.05; Figure 6) and appeared more potent than in chronic UP-infected animals receiving LPS at 7 days pre-delivery (*p* = 0.08; Figure 6).

**Figure 7:**
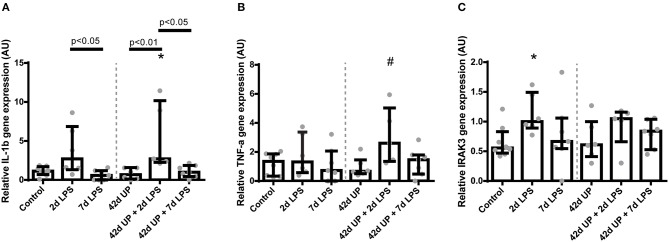
Relative mRNA levels of *IL-1*β **(A)**, *TNF-*α **(B)**, and *IRAK3* **(C)** in arbitrary unit (AU). **(A)** Increased *IL-1*β mRNA level in animals exposed to 2 days lipopolysaccharide (LPS) and *Ureaplasma parvum* (UP) prior to 2 days LPS. *p < 0.05 compared to the control. **(B)** Increased *TNF-*α mRNA level in animals exposed to UP prior to 2 days LPS. ^#^*p* = 0.07 compared to the control. **(C)** Increased *IRAK3* mRNA level in animals exposed to 2 days LPS. *p < 0.05 compared to the control.

Examination of underlying cytokine levels revealed increased *IL-1*β mRNA levels only in the uninfected or chronic UP-infected animals when LPS was administered 2 days pre-delivery (*p* < 0.05 compared to the 7 days LPS group, *p* < 0.01 compared to the 42 days UP group, *p* < 0.05 compared to the 42 days UP + 7 days LPS group, and *p* < 0.05 compared to the control, respectively; Figure 7A), whereas *IL-1*β mRNA levels had dropped to baseline again if LPS was administered 7 days pre-delivery (Figure 7A). *IL-6* and *IL-10* mRNA levels were not altered (data not shown), and the only group showing apparent *TNF-*α mRNA level elevation was that of the chronic UP-infected animals additionally receiving LPS 2 days pre-delivery (*p* = 0.07; Figure 7B).

*IRAK3* mRNA levels were increased significantly only in animals exposed to 2 days of LPS alone compared to the control (*p* < 0.05; Figure 7C).

### ENS Alterations in the Terminal Ileum Due to Chronic IA UP Exposure

**Figure 8:**
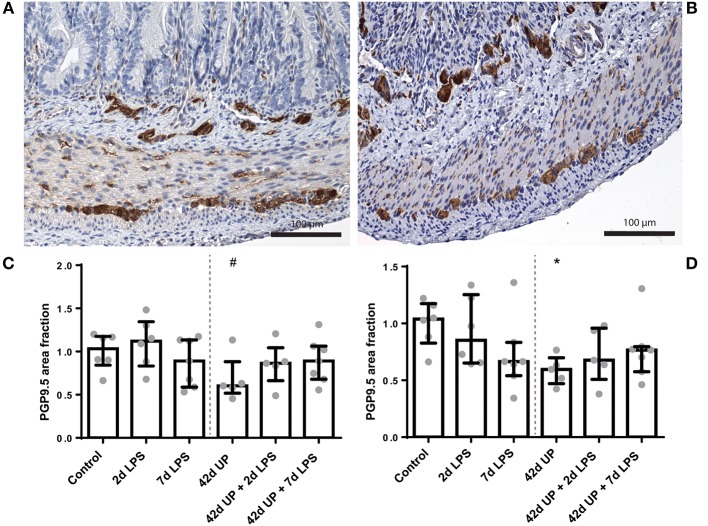
Representative images of PGP9.5 immunoreactivity in the submucosal and myenteric plexus of the control **(A)** and *Ureaplasma parvum* (UP) **(B)** groups. Area fraction of PGP9.5 in the submucosal plexus **(C)** and myenteric plexus **(D)** as fold increase over the control value. **(C)** PGP9.5-positive surface area tended to be decreased in the submucosal plexus of animals exposed to UP. ^#^*p* = 0.08 compared to the control. **(D)** Decreased PGP9.5-positive surface area in the myenteric plexus of animals exposed to UP. *p < 0.05 compared to the control.

The PGP9.5-positive surface area in the submucosal plexus tended to be decreased in animals chronically infected for 42 days with UP compared to the control (*p* = 0.08; Figure 8). Similarly, chronic UP-infected animals had a diminished PGP9.5-positive surface area in the myenteric plexus (*p* < 0.05; Figure 8). Doublecortin-positive surface areas were not altered in either the submucosal or the myenteric plexus (data not shown).

**Figure 9:**
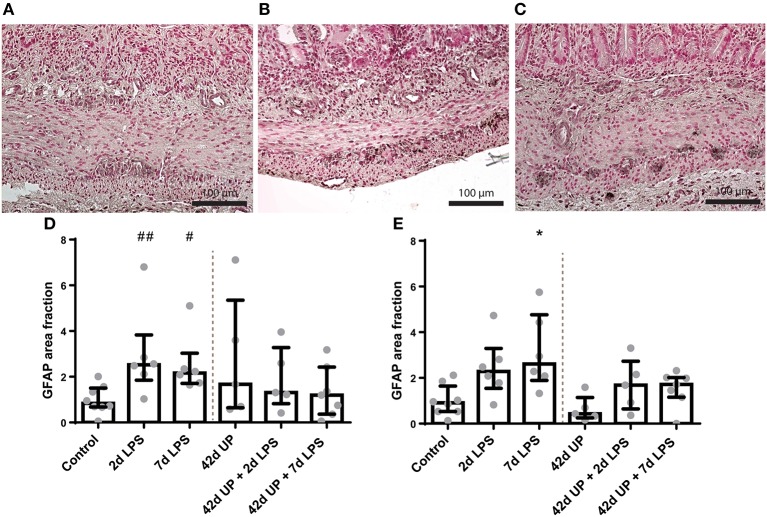
Representative images of glial fibrillary acidic protein (GFAP) immunoreactivity in the submucosal and myenteric plexus of the control **(A)**, 2 days lipopolysaccharide (LPS) **(B)**, and 7 days LPS **(C)** group. Area fraction of GFAP in the submucosal plexus **(D)** and myenteric plexus **(E)** as fold increase over the control value. **(D)** The GFAP-positive surface area tended to be increased in the submucosal plexus of animals exposed to 2- and 7 days LPS. ^##^*p* = 0.07, ^#^*p* = 0.09 compared to the control. **(E)** Increased GFAP-positive surface area in the myenteric plexus of animals exposed to 7 days LPS. *p < 0.05 compared to the control.

In the submucosal plexus, the GFAP-positive surface area tended to be increased in groups receiving LPS either 2 or 7 days pre-delivery compared to the control (*p* = 0.07 and *p* = 0.09; Figure 9), while in the myenteric plexus, the GFAP-positive surface area was only increased in the group receiving LPS 7 days pre-delivery compared to the control (*p* < 0.05; Figure 9). For both of these regions, concomitant chronic infection by UP appeared to mute these effects.

**Figure 10:**
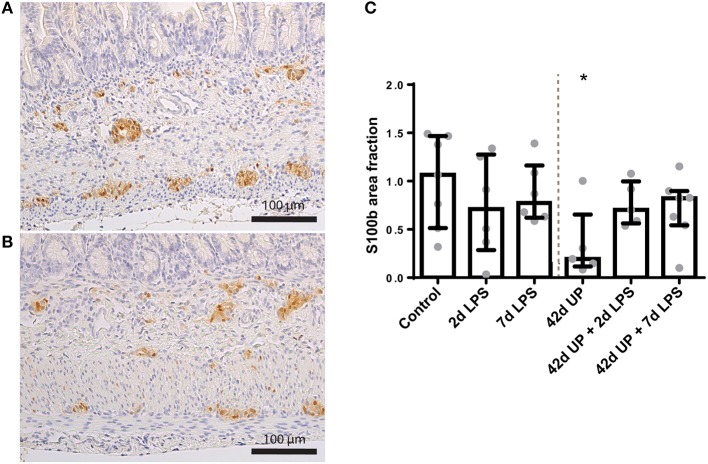
Representative images of S100β immunoreactivity in the submucosal and myenteric plexus of the control **(A)** and *Ureaplasma parvum* (UP) group **(B)**. Area fraction of S100β in the myenteric plexus **(C)** as fold increase over the control value. **(C)** The S100β-positive surface area was decreased in the myenteric plexus of animals exposed to UP. *p < 0.05 compared to the control.

S100β-positive surface areas were unaltered in the submucosal plexus for all conditions (data not shown), while in the myenteric plexus, the S100β-positive surface area was significantly decreased in the chronic UP-infected group compared to the control (*p* < 0.05; Figure 10), but this effect appeared to be counteracted by acute stimulation by LPS at either 2 or 7 days pre-delivery. No differences in *nNOS* and *CHAT* expression were observed between the groups (data not shown).

## Discussion

In this study, we investigated the effect of acute, chronic, and combined microbial exposure as an antenatal infectious trigger (chorioamnionitis) on the mucosa, submucosa, and ENS of the terminal ileum of premature lambs.

Both acute LPS and chronic UP exposure caused mucosal inflammation and injury to the terminal ileum. Although the inflammatory signature differed between these groups, mucosal injury was not aggravated in the combined exposure groups. Prenatal IA exposure to 7 days of LPS and to chronic 42 days infection by UP both provoked an influx of neutrophils (MPO-positive cells) in the intestine. In contrast, T cell (CD3-positive cells) numbers remained unaltered in the chronic UP and 2 days LPS groups compared to the control group, but were increased in the UP + 2 days LPS-exposed animals, indicating a potential synergistic effect of both inflammatory stimuli in inducing an adaptive mucosal immune response. We observed a similar effect in the submucosa: while either LPS exposure or UP infection induced innate immune changes in the ileum, T cell alterations only occurred in the presence of combined UP and LPS exposure. Based on the current findings, we can only speculate on the mechanism behind this synergistic effect. In previous *in vitro* studies, signaling via Toll-like receptors (*TLR*) 1, 2, and 6 by *Ureaplasma* spp. increased LPS-mediated inflammation (33, 34). Additionally, *TNF-*α mRNA levels tended to be increased in the UP-infected animals receiving LPS 2 days pre-delivery, while *TNF-*α levels were not increased upon single exposure to either UP or LPS alone. In contrast, no synergistic effect of UP and LPS exposure on intestinal *IL-1*β mRNA levels was found in the current study. This is supported by our *IRAK3* mRNA findings, a negative regulator of *TLR* signaling (35), which remained unaltered in combined exposure of UP-infected animals to LPS. Based on these joined findings, it is tempting to speculate that UP + LPS induced the upregulation of cell adhesion molecules and, consequently, temporarily increased diapedesis, which could at least in part be responsible for the observed increase in CD3-positive cells. The latter suggestion is supported by previous *in vitro* findings showing enhanced endothelial protein expression of the cell adhesion molecule *CXCR7* following co-incubation with LPS and UP, which was not observed in independently UP- or LPS-exposed cells (36).

Interestingly, other ovine studies have reported a suppressive immune effect in the premature lung and brain after chronic UP exposure prior to acute LPS exposure (18, 19). Taken together, these data show that cells might be sensitized, preconditioned, or remain unaffected following chronic UP infection, indicating organ-dependent responses. The mechanisms responsible for organ-specific effects of a second-hit chorioamnionitis remain to be elucidated.

The ENS closely interacts with intestinal immune cells (37). As such, ENS alterations can both result from intestinal inflammation and modulate it (38, 39). In this study, the most evident signs of ENS alterations were seen after chronic UP infection, which caused a reduced PGP9.5-positive surface area in both plexuses, likely representing a loss of enteric neurons. Alternatively, this might represent a loss of PGP9.5 positivity by enteric neurons. The doublecortin-positive (immature neurons) surface area in chronically UP-infected animals was unchanged, indicating that a decrease of mature neurons is responsible for the observed neuronal cell loss. As the period between 10 and 18 weeks of gestation is considered to be of paramount importance for both morphological and functional maturation of the ENS (40, 41), one might assume that the timing of our inflammatory challenge during this vulnerable second trimester is the key determinant for the observed effects rather than the nature of the microbial trigger. The loss of enteric neurons in the myenteric plexus following chronic UP infection coincides with a reduced S100β-positive surface area, suggesting a reduced number of enteric glial cells. However, a reduction of S100β immunoreactivity within glial cells could be involved in the observed effect as well. Enteric glial cells are known to contribute to neuronal maintenance, survival, and function (42). Interestingly, the S100β-positive surface area was less reduced in the groups exposed to an additional LPS challenge in combination with chronic UP infection, and the median of the PGP9.5-positive surface area was higher in these groups. In support, previous studies have shown that enteric glial cells are capable of generating enteric neurons in response to injury (43, 44), indicating that glial cells could be the driving cells behind the loss or gain of neurons in our model. As a hallmark of their high level of cellular plasticity (45), enteric glia can respond to inflammatory cues and ENS damage by alternating their morphology and the expression of key proteins such as GFAP, in a process similar to reactive astrogliosis (46, 47). In this study, GFAP immunoreactivity was increased in both plexuses in the LPS-exposed animals, indicating that a glial response is induced by intestinal inflammation (48). An enteric glial cell response was not detected in chronic UP-infected animals despite signs of intestinal inflammation, suggesting normalization of the GFAP levels within this period. Interestingly, pre-conditioning through chronic UP infection prevented GFAP upregulation in response to the overlapping second challenge with LPS in the glial cells in both plexuses, as no altered GFAP immunoreactivity was seen following subsequent IA LPS exposure. Whether this is solely protective or can contribute to the ENS damage seen in chronic UP exposure is unclear, as activation of enteric glia in the context of intestinal inflammation has been described to be both destructive (49) and potentially neuroregenerative (50). We may conclude from the aforementioned findings that enteric glial cells are already able to react to inflammatory cues prenatally. Importantly, our results suggest that these cells play an important role in neuronal survival and neurogenesis in the intrauterine setting.

At present, the postnatal consequences of the detected loss of mature neurons and glial cells following UP exposure in the second trimester remain unknown. A similar decrease in enteric neurons has been described in models of experimental colitis, which show that neuronal loss persists after recovery of inflammation (51) and is accompanied by a decreased colonic motility (52). Based on these combined findings, it is likely that the observed changes *in utero* will result in ENS dysfunction postnatally.

Interestingly, several studies describe intestinal changes in patients with acute NEC that are similar to those found after chronic UP infection, namely, loss of both enteric neurons (25–27, 53) and glial cells (25–27). Moreover, it has been suggested that ablation of enteric glial cells may be an upstream target of NEC pathology (54). A potential causal role of the ENS in NEC pathophysiology is further supported by a rat study in which increased NEC survival and intestinal motility was associated with improvement of ENS changes, including an increase in enteric neurons (27). Collectively, our findings form a novel mechanistic explanation for the reported association of chorioamnionitis and NEC.

A limitation of this study is that it only enables us to study the effects of UP and LPS exposure at one time point, preventing us from dissecting the role of the different inflammatory triggers (LPS and UP) of inflammation duration (acute and chronic). In addition, the group sizes are small, which is an inherent shortcoming of the translational ovine model used.

In summary, an acute inflammatory stimulus with LPS or a chronic inflammatory stimulus with UP causes intestinal injury and inflammation in the mucosal and submucosal layers of the gut. Combined overlapping microbial exposure does not aggravate injury of the terminal ileum. Most importantly, chronic UP infection causes structural ENS alterations characterized by PGP9.5 and S100β immunoreactivity loss. Whether the observed ENS alterations result in functional abnormalities after birth remains to be elucidated. However, the observed changes *in utero* correspond with findings in neonates with NEC, which underlines the concept that NEC pathophysiology may already have its origin *in utero*.

## Data Availability Statement

The datasets generated for this study are available on request to the corresponding author.

## Ethics Statement

The animal study was reviewed and approved by The Animal Ethics Committee of the University of Western Australia (Perth, Australia).

## Author Contributions

CH, IL, MH, WG, and TW conceived the original idea. MS, HU, SS, OS, MB, MP, JN, AJ, and MK designed the *in vivo* model and performed the animal experiments. CH carried out the laboratory analyses with the support from NR, LK, and GR. CH, IL, MH, KL, VM, WB, BK, WG, and TW contributed to the interpretation of the results. CH and IL wrote the manuscript with the input from all authors. WG and TW supervised the project. All authors contributed to manuscript revision, read, and approved the submitted version.

### Conflict of Interest

The authors declare that the research was conducted in the absence of any commercial or financial relationships that could be construed as a potential conflict of interest. The handling editor declared a past co-authorship with several of the authors TW, BK.

## Abbreviations

AU: Arbitrary unit
BSA: Bovine serum albumin
CD3: Cluster of differentiation 3
CCU: Color-changing units
*CHAT*: Choline acetyltransferase
ENS: Enteric nervous system
FCS: Fetal calf serum
FIRS: Fetal inflammatory response syndrome
*GAPDH*: Glyceraldehyde 3-phosphate dehydrogenase
GFAP: Glial fibrillary acidic protein
IA: Intra-amniotic
IBD: Inflammatory bowel disease
IL: Interleukin
*IRAK3*: Interleukin-1 receptor-associated kinase 3
LPS: Lipopolysaccharide
MPO: Myeloperoxidase
NEC: Necrotizing enterocolitis
NGS: Normal goat serum
*nNOS*: Neuronal nitric oxide synthase
PBS: Phosphorylated buffer saline
PGP9.5: Protein gene product 9.5
*PPIA*: Peptidylprolyl isomerase A
*RPS15*: Ribosomal protein S15
Spp: Species
*TLR*: Toll-like receptor
*TNF-*α: Tumor necrosis factor alpha
UP: *Ureaplasma parvum*.
