# Corrigendum: Chronic Intra-Uterine *Ureaplasma parvum* Infection Induces Injury of the Enteric Nervous System in Ovine Fetuses

**DOI:** 10.3389/fimmu.2020.00672

**Published:** 2020-04-15

**Authors:** Cathelijne Heymans, Ilse H. de Lange, Matthias C. Hütten, Kaatje Lenaerts, Nadine J. E. de Ruijter, Lilian C. G. A. Kessels, Glenn Rademakers, Veerle Melotte, Werend Boesmans, Masatoshi Saito, Haruo Usuda, Sarah J. Stock, Owen B. Spiller, Michael L. Beeton, Matthew S. Payne, Boris W. Kramer, John P. Newnham, Alan H. Jobe, Matthew W. Kemp, Wim G. van Gemert, Tim G. A. M. Wolfs

**Affiliations:** ^1^Department of Surgery, NUTRIM School of Nutrition and Translational Research in Metabolism, Maastricht University, Maastricht, Netherlands; ^2^Department of Pediatrics, School for Oncology and Developmental Biology (GROW), Maastricht University, Maastricht, Netherlands; ^3^Neonatology, Department of Pediatrics, Maastricht University Medical Center, Maastricht, Netherlands; ^4^Neonatology, Department of Pediatrics, University Hospital Aachen, Aachen, Germany; ^5^Neonatology, Department of Pediatrics, University Children's Hospital Würzburg, Würzburg, Germany; ^6^Department of Pathology, School for Oncology and Developmental Biology (GROW), Maastricht University Medical Center, Maastricht, Netherlands; ^7^Biomedical Research Institute (BIOMED), Hasselt University, Hasselt, Belgium; ^8^Division of Obstetrics and Gynecology, University of Western Australia, Perth, WA, Australia; ^9^Center for Perinatal and Neonatal Medicine, Tohoku University Hospital, Sendai, Japan; ^10^Usher Institute, University of Edinburgh, Edinburgh, United Kingdom; ^11^Division of Infection and Immunity, School of Medicine, Cardiff University, Cardiff, United Kingdom; ^12^Cardiff School of Sport and Health Sciences, Cardiff Metropolitan University, Cardiff, United Kingdom; ^13^Division of Neonatology/Pulmonary Biology, The Perinatal Institute, Cincinnati Children's Hospital Medical Center, University of Cincinnati, Cincinnati, OH, United States; ^14^School of Veterinary and Life Sciences, Murdoch University, Perth, WA, Australia; ^15^Pediatric Surgery, Department of Surgery, Maastricht University Medical Center, Maastricht, Netherlands; ^16^Department of Surgery, University Hospital Aachen, Aachen, Germany; ^17^Department of Biomedical Engineering (BMT), School for Cardiovascular Diseases (CARIM), Maastricht University, Maastricht, Netherlands

**Keywords:** *Ureaplasma parvum*, intra-amniotic infection, chorioamnionitis, enteric nervous system, sheep, preterm birth, necrotizing enterocolitis

Michael L. Beeton was not included as an author in the published article. The corrected Author Contributions Statement appears below.

## Author Contributions

CH, IL, MH, WG, and TW conceived the original idea. MS, HU, SS, OS, MB, MP, JN, AJ, and MK designed the *in vivo* model and performed the animal experiments. CH carried out the laboratory analyses with the support from NR, LK, and GR. CH, IL, MH, KL, VM, WB, BK, WG, and TW contributed to the interpretation of the results. CH and IL wrote the manuscript with the input from all authors. WG and TW supervised the project. All authors contributed to manuscript revision, read, and approved the submitted version.

The authors apologize for this error and state that this does not change the scientific conclusions of the article in any way. The original article has been updated.

